# A genome-wide association study identifies genetic determinants of hemoglobin glycation index with implications across sex and ethnicity

**DOI:** 10.3389/fendo.2024.1473329

**Published:** 2024-10-28

**Authors:** John S. House, Joseph H. Breeyear, Farida S. Akhtari, Violet Evans, John B. Buse, James Hempe, Alessandro Doria, Josyf C. Mychaleckyi, Vivian Fonseca, Mengyao Shi, Changwei Li, Shuqian Liu, Tanika N. Kelly, Daniel Rotroff, Alison A. Motsinger-Reif

**Affiliations:** ^1^ Biostatistics and Computational Biology Branch, National Institute of Environmental Health Sciences, National Institutes of Health, Durham, NC, United States; ^2^ Division of Endocrinology, Department of Medicine, University of North Carolina School of Medicine, Chapel Hill, NC, United States; ^3^ Department of Pediatrics, Louisiana State University School of Medicine, New Orleans, LA, United States; ^4^ Section on Genetics and Epidemiology, Joslin Diabetes Center and Department of Medicine, Harvard Medical School, Boston, MA, United States; ^5^ Center of Public Health Genomics, School of Medicine, University of Virginia, Charlottesville, VA, United States; ^6^ Section of Endocrinology, School of Medicine, Tulane University, New Orleans, LA, United States; ^7^ Department of Epidemiology, Tulane University School of Public Health and Tropical Medicine, New Orleans, LA, United States; ^8^ Department of Health Policy and Management, Tulane University School of Public Health and Tropical Medicine, New Orleans, LA, United States; ^9^ Tulane University Translational Science Institute, New Orleans, LA, United States; ^10^ Department of Quantitative Health Sciences, Lerner Research Institute, Cleveland Clinic, Cleveland, OH, United States; ^11^ Endocrinology and Metabolism Institute, Cleveland Clinic, Cleveland, OH, United States; ^12^ Cleveland Clinic Lerner College of Medicine, Case Western Reserve University, Cleveland, OH, United States

**Keywords:** hemoglobin glycation index, HGI, HbA1c, ACCORD, ARIC, GWAS, genome-wide association study

## Abstract

**Introduction:**

We investigated the genetic determinants of variation in the hemoglobin glycation index (HGI), an emerging biomarker for the risk of diabetes complications.

**Methods:**

We conducted a genome-wide association study (GWAS) for HGI in the Action to Control Cardiovascular Risk in Diabetes (ACCORD) trial (*N* = 7,913) using linear regression and additive genotype encoding on variants with minor allele frequency greater than 3%. We conducted replication analyses of top findings in the Atherosclerosis Risk in Communities (ARIC) study with inverse variance-weighted meta-analysis. We followed up with stratified GWAS analyses by sex and self-reported race.

**Results:**

In ACCORD, we identified single nucleotide polymorphisms (SNPs) associated with HGI, including a peak with the strongest association at the intergenic SNP *rs73407935* (7q11.22) (*P* = 5.8e−10) with a local replication in ARIC. In black individuals, the variant *rs10739419* on chromosome 9 in the Whirlin (*WHRN*) gene formally replicated (meta-*P* = 2.2e−9). The SNP-based heritability of HGI was 0.39 (*P*< 1e−10). HGI had significant sex-specific associations with SNPs in or near *GALNT11* in women and *HECW2* in men. Finally, in Hispanic participants, we observed genome-wide significant associations with variants near *USF1* and *NXNL2/SPIN1*.

**Discussion:**

Many HGI-associated SNPs were distinct from those associated with fasting plasma glucose or HbA1c, lending further support for HGI as a distinct biomarker of diabetes complications. The results of this first evaluation of the genetic etiology of HGI indicate that it is highly heritable and point to heterogeneity by sex and race.

## 1 Introduction

Hemoglobin A1c (HbA1c) provides a more stable estimate of glycemic control over a 2- to 3-month period than point-estimate fasting plasma glucose (FPG). HbA1c is routinely used in diabetes diagnosis and treatment and is correlated with the risk of complications from diabetes. Collectively, HbA1c and FPG offer a nuanced view of an individual’s disease status. However, there is considerable heterogeneity in HbA1c, even among individuals with similar FPG or mean glucose levels. Because HbA1c is not a one-size-fits-all indicator of overall blood glucose control, the hemoglobin glycation index (HGI), which quantifies FPG‐independent biological variation in HbA1c in individuals with diabetes, may be important as an additional biomarker of clinical management/drug treatment outcomes. HGI is the difference between observed HbA1c and HbA1c predicted from other measures of glycemia (e.g., FPG) using linear regression. Studies of HGI, the glycation gap (1), and individuals with higher HbA1c values than those predicted by blood glucose reflect similar unexplained phenotypic variation in the temporal assessment of glycemic control predicted by a point estimate. Variation in HbA1c has been observed in individuals with and without diabetes and may be influenced by biological factors in addition to glucose, including erythrocyte lifespan, intracellular glucose concentration, intracellular or extracellular pH, deglycating enzyme activity, lipid peroxides, inorganic phosphates, hemoglobin oxygenation status, and inflammation (2–4). A recent landmark study in the Million Veterans Program highlighted that unexplained variation in HbA1c due to FPG contributes to increased disease progression at the time of diagnosis and increased risk of diabetes complications in men of African ancestry (5).

Previous studies have demonstrated that HGI can be used to identify individuals with type 2 diabetes at risk for multiple diabetes-related comorbidities and outcomes before the onset of other clinical symptoms (6). In a meta-analysis of 37,280 individuals with type 2 diabetes, those with high HGI had an increased risk of cardiovascular-related complications and death (7). HGI has been associated with the risk of retinopathy and nephropathy in individuals with type 1 diabetes and with the risk of coronary heart disease in individuals with type 2 diabetes (7) and those with normal glucose metabolism (8). As reviewed by Zhang et al. (7), in individuals with diabetes, high HGI calculated using either FPG or mean blood glucose (from patient meters or 1-day profile sets collected before and after meals) has been associated with a higher risk of hypertension, microvascular complications, hypoglycemia, and cardiovascular disease. In individuals without diabetes, high HGI has been associated with increased coronary artery calcification (9), carotid atherosclerosis (10), hepatic steatosis (11), kidney dysfunction (12), and inflammation and obesity (13, 14). Despite these associations, it has been suggested that because HGI is strongly associated with HbA1c, it should not be used as an independent predictor of microvascular complications (15). Studies have also reported increased HGI in black individuals and women (16). This evidence indicates that HGI reflects complexities in glucose control and the risk of chronic vascular disease in individuals both with and without diabetes.

HGI is a promising biomarker to determine the risk of diabetes comorbidities. Understanding the genetic etiology of this trait is an important goal and could provide insight into druggable targets and guide precision medicine approaches to treatment. While the genetics of HbA1c and other related traits have been extensively studied (17–21), to the authors’ knowledge, no studies have directly examined the genetics of HGI. To address this gap, we performed a genome-wide association study (GWAS) of HGI using data from the Action to Control Cardiovascular Risk in Diabetes (ACCORD) trial and replicated our findings using data from the Atherosclerosis Risk in Communities (ARIC) study, an independent cohort. In the spirit of discovery, we conducted stratified analyses by both genetic ancestry and biological sex.

## 2 Methods

### 2.1 Phenotype definition

We derived HGI by regressing FPG on HbA1c in ACCORD as previously reported (22). For the overall cohort and the 7,913 individuals genotyped in this study, the fitted line equates to: $\hat{\text{HbA}1\text{c}}=\;6.8\;+\;0.009\times \text{FPG}\left[\frac{\text{mg}}{\text{dL}}\right]$ . We calculated the individual HGI from the residual (i.e., HbA1c_i_ − $\hat{\text{HbA}1{\text{c}}_{i}}$ ). For GWAS of HbA1c and FPG separately, and to calculate HGI, we used baseline values for HbA1c and FPG.

### 2.2 Genotyping

We performed genotyping using the HumanOmniExpressExome-8 v1.0 and Affymetrix Axiom Biobank1 arrays. Genotyping and imputation details are included in the **Supplementary Material** and have been previously described.

### 2.3 Genome-wide association study

Details of the ACCORD genotyping can be found in the **Supplementary Material**. In brief, of the 10,250 ACCORD participants, 8,174 were genotyped across two platforms. Extensive QC, merging, and imputation were conducted and had been previously described (23, 24) and resulted in 8,054 participants with genetic data. After genotyping QC and phenotyping with the requisite FPG and HbA1c baseline values, 7,913 individuals were used in this GWAS. We conducted GWAS using ACCORD data to determine single nucleotide polymorphisms (SNPs) associated with baseline HGI using linear regression in six strata, namely, all participants, all men, all women, and white, black, and Hispanic individuals. To address population stratification, we conducted principal component analysis (PCA) using EIGENSTRAT v4.2 (25) and incorporated the principal components as model covariates. For each stratum, we repeated variable selection and PCA to address potential confounding. Some covariates were forced based on the results of previous studies and domain expertise, and others were based on backward selection using Bayesian information criteria. **Supplementary Table 1** summarizes the selected covariates for each stratum, which include principal components, sex, years with type 2 diabetes, BMI, and other covariates. **Supplementary Table 2** lists all the covariates considered for variable selection in ACCORD.

We used linear regression implemented in PLINK (26) (v1.9; www.cog-genomics.org/plink/1.9/) to test associations between phenotypes, covariates, and single common variants [minor allele frequency (MAF) > 3%]. We used additive encoding for genotyped variants, where ${ɡ}_{i}\in \left\{0,1,2\right\}$ is the number of minor alleles for the i th individual. We implemented linear regression in the R programming language for imputed variants, where ${ɡ}_{i}=p_{1}\left(Aa\right)+2p_{i}\left(aa\right)$ is the dosage score computed from the posterior probabilities for genotypes Aa and aa . Samples were genotyped in two sets that were determined based on participant consent. For SNPs that were genotyped only in set 1 and imputed in set 2, we calculated association tests for each set separately and combined the results by meta-analysis using PLINK. SNPs with *P<*5e−8 were considered to have genome-wide significance, and those with *P<*5e−6 were considered to have suggestive significance. We report GWAS results for all participants, for all-male and all-female strata, and for white, black, and Hispanic individuals. We calculated SNP-based heritability with GCTA64 (27) (version 1.94.0; reml-grm and reml-alg 1 options), incorporating model covariates as described above.

### 2.4 Replication analysis in ARIC

Between 1987 and 1989, the ARIC prospective cohort study enrolled 15,792 adults aged 45 to 64 years from four U.S. communities (Forsyth County, NC; Jackson, MS; suburbs of Minneapolis, MN; and Washington County, MD) to examine the etiology and predictors of cardiovascular disease. The ARIC study design and methods are described in detail elsewhere (28). Notably, there are substantial differences in the ACCORD and ARIC cohorts in terms of the age of participants, mean HbA1c, and diabetes treatments, all of which have been associated with differences in HGI (see **Table 1**). This heterogeneity between cohorts minimizes potential associations due to bias. We downloaded the genome-wide autosomal SNP data available for 8,620 white ARIC participants from the database of Genotypes and Phenotypes (dbGaP). SNPs were genotyped using the Affymetrix 6.0 array and underwent previous sample quality control that included the removal of related pairs, duplicates, and sex misclassifications. We performed further preimputation quality control, additionally removing samples with a call rate<80% and SNPs with Hardy–Weinberg equilibrium (HWE) *P*<1e−6, missingness >10%, or MAF<3%. After filtering, 703,117 SNPs remained for genotype imputation. We conducted imputation from the *All*-ancestry panel of the 1000 Genome Phase III Integrated Release Version 5 using MiniMac software. After imputation, we removed SNPs with *r* ^2^<0.30, MAF<1%, or HWE *P*<1 × 10^−6^ and retained 10,008,913 SNPs. Individuals selected for replication consisted of individuals with both FPG and HbA1c data (assays conducted at visit 5 follow-up ending in 2013), as well as genome-wide genotyping data (*N* = 3,741; 2,101 F/1,640 M; 3,160 EUR/581 AFR).

**Table 1:** Demographics of the ACCORD and ARIC participants.

	ACCORD (F)	ACCORD (M)	ACCORD	ARIC
Participants—*N* (%)	3,124 (39.48%)	4,789 (60.52%)	7,913 (100.00%)	3,741
HGI—mean (SD)	0.06 (0.89)	−0.04 (0.88)	0.00 (0.88)	0.00 (0.59)
HbA1c—mean (SD)	8.33 (1.01)	8.25 (0.98)	8.28 (1.00)	5.92 (0.81)
FPG—mean (SD)	173.15 (52.58)	176.62 (53.76)	175.25 (53.32)	113.05 (27.64)
BMI—mean (SD)	33.31 (5.78)	31.69 (5.03)	32.33 (5.40)	28.51 (5.40)
Age—mean (SD)	62.57 (6.30)	62.88 (6.57)	62.76 (6.47)	76.66 (5.12)

We assessed discovery variants from the ACCORD data with *P<*5e−6 for the overall cohort (*N* = 7,913) and strata for white and black participants (*n* = 5,085 and *n* = 1,378, respectively) from the ARIC data (**Supplementary Table 3**). ARIC performed association tests for each discovery variant using linear regression with an additive genetic model, adjusting for age, sex, BMI, trial clinic center, and the first 11 principal components. We analyzed summary statistics from the discovery (ACCORD) and replication (ARIC) cohorts (**Supplementary Table 3**) and conducted an inverse-variance-weighted meta-analysis using METAL (https://genome.sph.umich.edu/wiki/METAL_Documentation).

In total, we tested for replication of discovery variants with *P*<5e−6 for the three strata looked up in ARIC (all individuals, white individuals, and black individuals). The number of independent loci in each stratum was 24, 9, and 15, respectively. We considered a discovery finding to be formally replicated when a genome-wide significant (*P*< 5e−8) sentinel discovery variant was also found in ARIC with the same direction of effect and a replication *P*<0.05/number of independent loci, or when any discovery variant at suggestive significance (5e−8< *P*< 5e−6) achieved genome-wide significance after meta-analysis of discovery and ARIC. We defined local replication when any variant in discovery with *P*<5e−6 was found in ARIC with the same direction of effect and with replication *P*<0.05/number of independent loci.

### 2.5 Post-GWAS analysis

All annotations were mapped to the GRCh37 assembly. We developed data visualizations using R version 4.10 and LocusZoom (http://locuszoom.org/). We identified expression quantitative trait loci (eQTL) variants using FIVEx (https://fivex.sph.umich.edu/). We assessed genetic colocalization with the eQTL signal using LocusFocus (https://locusfocus.research.sickkids.ca/). LocusFocus utilizes a simple sum method to identify the colocalization of genetic variation signal and expression (via GTEx) in tissue by gene combinations (29). We examined characterized genes within 150 kbp of the lead SNP and tissues related to the pancreas, vessel, muscle, and brain (*n* = 19). The Bonferroni-corrected *P*-values for genetic colocalization were as follows: rs73407935 chr7:67680375, 15 gene/tissues (combinations passed stage 1 and were tested using a simple sum statistic) within 150 kbp, adjusted *P* = 0.0033 (0.05/15).

We defined loci associated with HGI as non-overlapping genomic regions separated by *r* ^2^<0.05 from (suggestive) association signals with *P*<5e−6 (**Table 2**). Complete results for all strata (*P*< 5e−6) can be found in **Supplementary Table 6**, and all variants for all GWAS strata are available through the LocusZoom hyperlinks in **Supplementary Table 4**.

**Table 2:** Hemoglobin glycation index of GWAS top loci (*r* ^2^<0.05, *P*<5e−6).

CHR	rsID	Position	Effect allele	Other allele	MAF	Beta	SE	*P-*value	Nearest gene
7	*rs73407935*	67680375	T	A	0.05	0.21	0.03	5.82E−10	*LOC105375341* ^*^
9	*rs73651763*	92899799	T	C	0.08	−0.14	0.03	7.49E−08	*LOC286370*
13	*rs9515223*	111115480	C	T	0.41	−0.07	0.01	2.05E−07	*COL4A2-AS1*
12	*rs16920729*	126989892	G	T	0.06	−0.18	0.04	1.21E−06	*LOC100128554*
6	*rs4715882*	14266008	A	G	0.05	−0.16	0.03	1.35E−06	*LINC01108*
7	*rs10235320*	151729550	T	C	0.12	−0.12	0.03	1.57E−06	*GALNT11*
12	*rs56816182*	129939812	A	C	0.03	−0.23	0.05	2.09E−06	*TMEM132D*
5	*rs16878633*	60504602	T	C	0.05	−0.15	0.03	2.20E−06	*CTC-436P18.1*
4	*rs6816425*	76983992	C	T	0.43	−0.07	0.01	2.23E−06	*CXCL11*
20	*rs71197800*	5775884	C	CA	0.29	0.07	0.02	2.28E−06	*SHLD1*
16	*rs7202789*	65744774	C	T	0.05	−0.17	0.04	2.33E−06	Intergenic
8	*rs6995047*	17979181	A	G	0.22	0.08	0.02	2.37E−06	*ASAH1*
6	*rs9348372*	19268181	G	C	0.14	−0.10	0.02	2.40E−06	*LOC101928519*
17	*rs7217547*	27106872	G	C	0.24	−0.08	0.02	2.47E−06	*FAM222B*
12	*rs200619018*	101089571	A	AATAAAAAT	0.09	0.13	0.03	2.53E−06	*GAS2L3*
13	*rs1927011*	103229210	C	A	0.46	−0.07	0.01	2.56E−06	*TPP2*
15	*rs57818172*	41092197	G	T	0.34	0.07	0.01	2.63E−06	*ZFYVE19*
2	*rs16843980*	210833037	T	C	0.05	0.15	0.03	2.97E−06	*UNC80*
4	*rs111298912*	110987610	A	G	0.16	−0.09	0.02	3.03E−06	*ELOVL6*
3	*rs73166944*	151504851	C	T	0.07	0.13	0.03	3.12E−06	*AADAC*
5	*rs75217746*	78630051	A	G	0.06	−0.16	0.03	3.30E−06	*JMY*
15	*rs17686675*	70364562	G	A	0.39	0.07	0.01	4.53E−06	*MIR629*
7	*rs10264140*	6236374	C	G	0.10	−0.12	0.03	4.68E−06	*CYTH3*
1	*rs78331180*	47956855	T	C	0.04	0.18	0.04	4.98E−06	*FOXD2*

^*^ Locally replicated in the ARIC.

## 3 Results

### 3.1 HGI heritability

The SNP-based heritability (*h* ^2^) was 0.39 (*P<* 1e−10) for the combined ACCORD cohort (*N* = 7,913), 0.52 (*P*< 1e−10) for male participants (*n* = 4,769), and 0.51 (*P* = 4.64e−7) for female participants (*n* = 3,111), indicating that a substantial portion of the variability in HGI is due to underlying genetic factors. This high degree of heritability is common in complex genetic traits and supports the rationale for conducting a GWAS.

### 3.2 Discovery HGI GWAS

**Figure 1:**
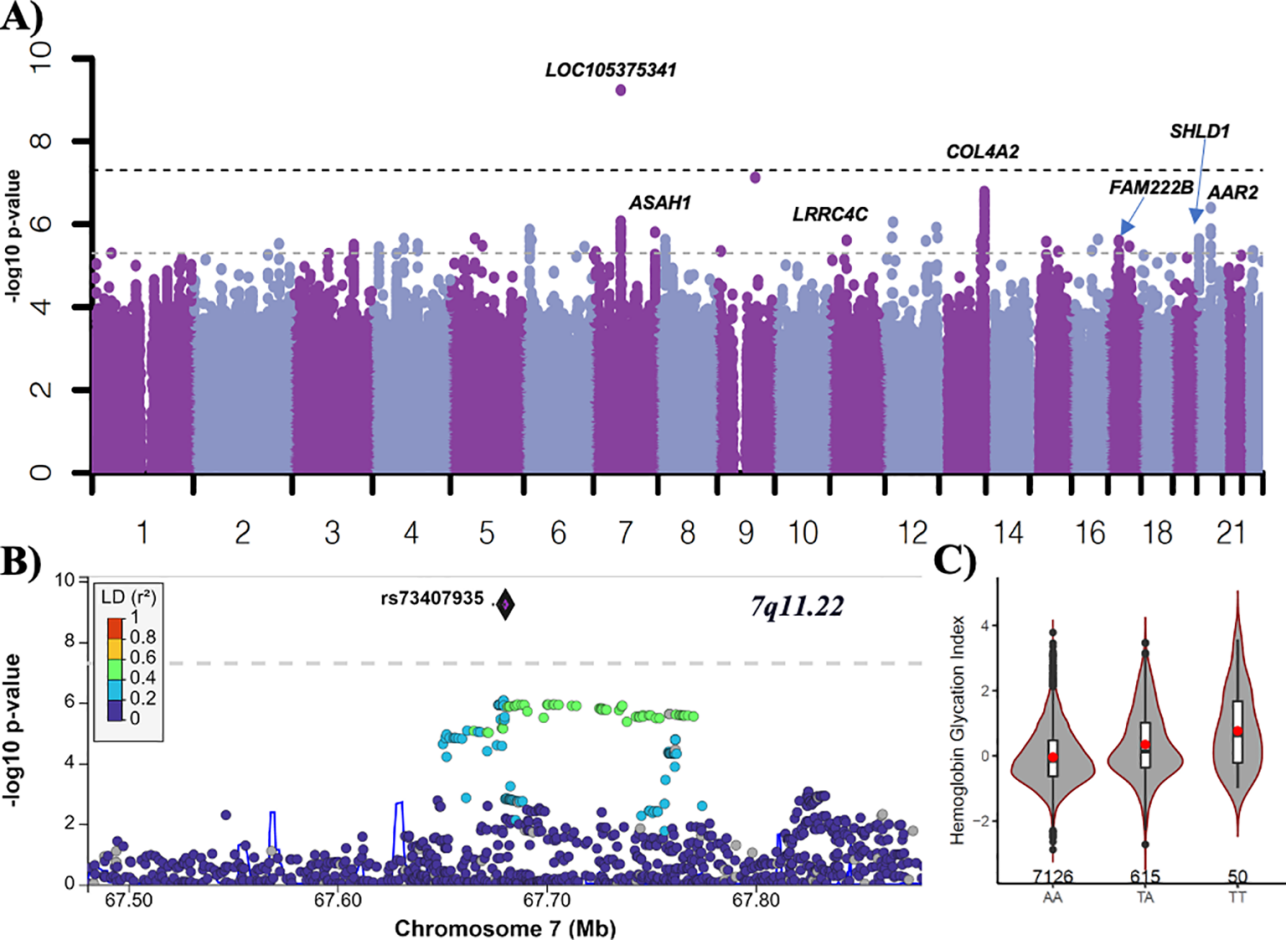
GWAS of HGI in ACCORD. **(A)** Manhattan plot for HGI for all subjects (*n* = 7,913). Variants with minor allele frequency >3% are ordered by chromosome (*X*-axis), and –log_10_ (*P-*values) are plotted (*Y*-axis). *P*-values are adjusted for baseline age, sex, BMI, treatment arm, principal components 1–3, number of years since diabetes diagnosis, and history of albuminuria. Dark dotted line: *P* = 5e−8; light dotted line *P =* 5e−6. **(B)** LocusZoom plot of the lead variant (*rs73407935*). **(C)** The allelic plot of the lead variant (*rs73407935*) showing HGI (*Y*-axis) against genotype (*X*-axis). A red dot represents the mean HGI response by genotype. The number of participants for each genotype is listed above the *X*-axis.

**Figure 2:**
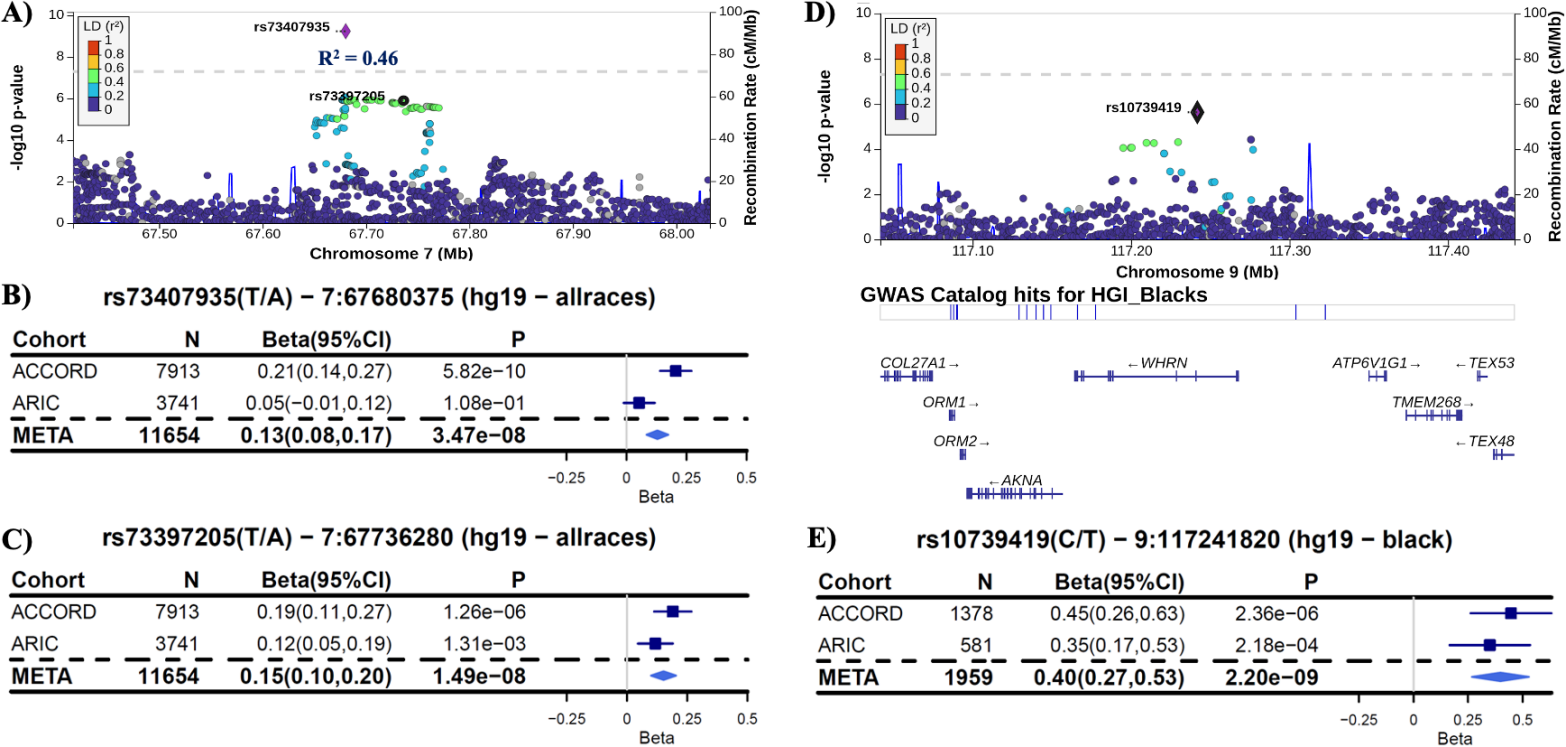
Replication in ARIC. **(A)** LocusZoom plot of the lead variant (*rs73407935*) with the formally replicated variant (*rs73397205*—*R* ^2^ = 0.46) annotated for all subjects. **(B)** Forest plot of the lead variant for discovery (ACCORD), replication (ARIC), and inverse-variance-weighted meta-analysis (META) estimates and 95% confidence intervals [coded allele (CA) = T]. **(C)** Forest plot of the variant in linkage disequilibrium with the lead SNP that formally replicated with *P*<0.0021 (0.05/24 unique non-correlated genomic regions examined in ARIC). Each copy of the coded allele (CA = A) results in an increase in HGI of approximately 0.15 for this variant. **(D)** LocusZoom plot of the variant *rs10739419*, which replicated in ARIC for black participants with meta-*P* reaching genome-wide significance. **(E)** Forest plot of *rs10739419* (CA = T).

In the ACCORD discovery, 24 independent loci were associated with HGI at genome-wide suggestive significance (**Table 2**). In the GWAS of HGI for all participants, the lead association with HGI was variation in the intergenic variant *rs73407935* (*LOC105375341*, *7q11*.*22*; *P* = 5.82e−10) on chromosome 7 (**Figures 1A, B**). The genetic dosage of the T allele was associated with increased HGI (**Figure 1C**). For replication in ARIC, we selected variants with suggestive genome-wide significance of *P*<5e−6 (see **Supplementary Table 3** for details). While the sentinel variant *rs73407935* was not formally replicated in ARIC (**Figures 2A, B**; same direction of effect, *P* = 0.11), we did have local replication with *rs73397205* in LD with the sentinel variant (*R* ^2^ = 0.46; **Figures 2A, C**; discovery *P =* 1.3e−06, replication *P* = 0.001) after correcting for 24 independent lookup loci in discovery. Furthermore, this variant reached genome-wide significance after meta-analysis (**Supplementary Table 5**).

**Figure 3:**
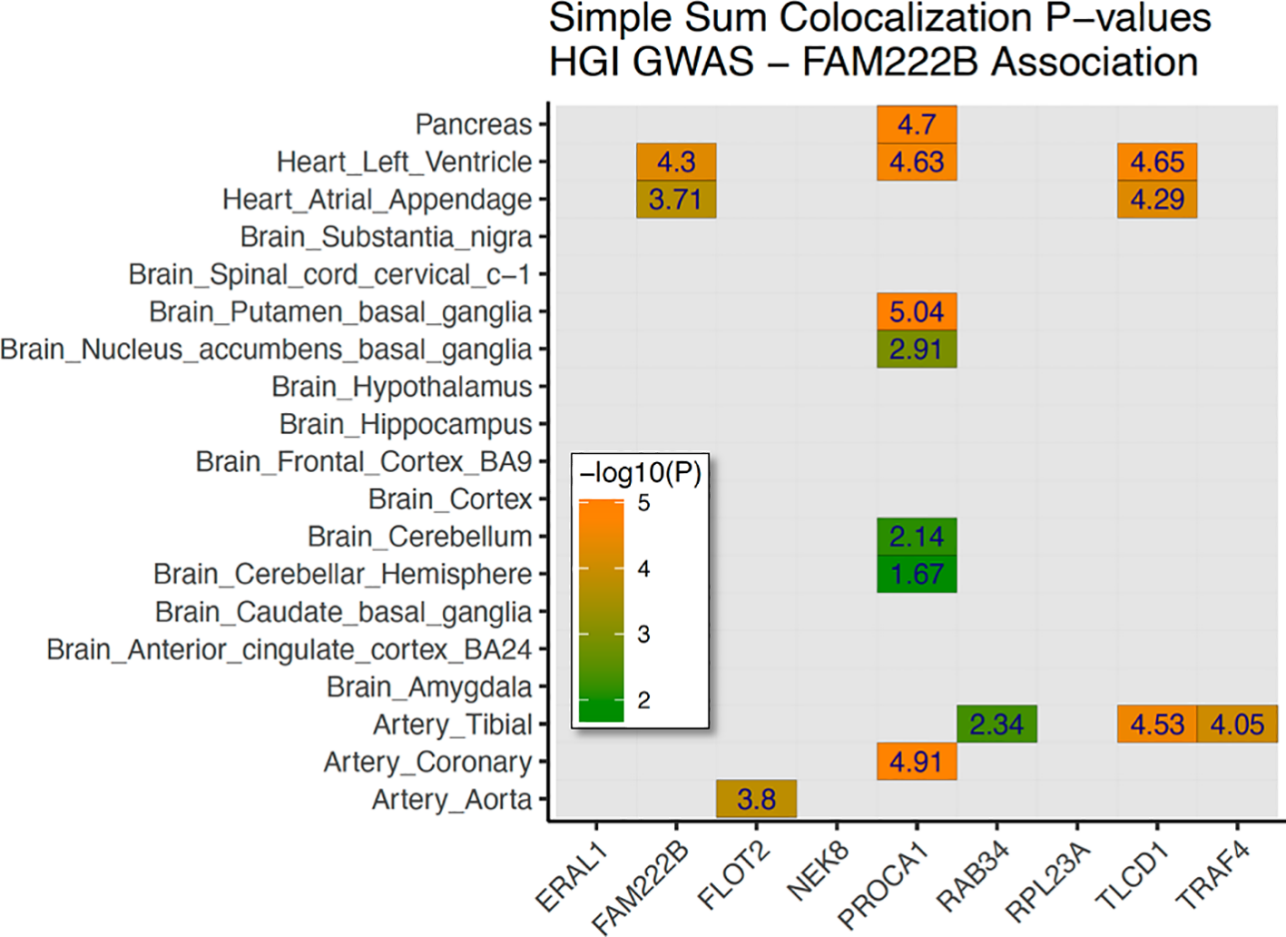
Genetic colocalization with gene expression—*FAM222B* discovery signal. Heatmap of 19 tissues (brain, heart, vessels, glands, and pancreas) with nine nearby genes tested for genetic correlation with GTEx data. In total, 15 tissue/gene combinations were selected for stage 2 colocalization. A family-wise Bonferroni-adjusted *P-*value of –log_10_(0.05/15) >2.477 was used to determine significance.

As the first GWAS of HGI, we also provided LocusZoom plots of genome-wide suggestive hits for variants in or near N-acylsphingosine amidohydrolase 1 (*ASAH1*), leucine-rich repeat containing 4 (*LRRC4*), NUT family member 2B (*NUTM2B*; formerly *FAM222B*), c (*SHLD1*), and collagen type iv alpha 2 chain (*COL4A2*) (**Supplementary Figures 1A–G**). Meta-analysis with ARIC for 178 overlapping variants with *P*<5e−6 demonstrated a consistent direction of effect in 150 variants (**Supplementary Table 5**). The two most significant variants in *FAM222B* were also strong eQTLs—*rs7217547* and *rs12943388* (**Supplementary Figures 1C–E**). As these variants are highly significant eQTLs associated with the regulation of expression of multiple genes in pancreatic and brain tissues and immune cell populations, we conducted a formal expression colocalization test with the LocusFocus online web tool (see the *Methods* section for details), which revealed that the *FAM222B* discovery locus was a strong eQTL after Bonferroni correction for 15 gene/tissue combinations. A strong colocalization was observed between genetic variation in the *FAM222B* locus and the expression of FAM222B in cardiac tissue; FLOT2 in aortic tissue; PROCA1 in pancreatic, cardiac, and brain tissue; TLCD1 in cardiac and arterial tissue; and TRAF4 in arterial tissue (**Figure 3**).

### 3.3 Stratified analysis

Because of the need to treat sex as a biological variable, and to further conduct valuable science in underrepresented populations, we performed stratified GWAS by self-reported race for white, black, and Hispanic participants and by sex for the overall analysis. **Supplementary Figure 2** displays the stacked Manhattan plot for HGI in combined/male/female strata with sex-specific associations for HGI. In women of all self-reported races, HGI was associated with genome-wide significant variants in polypeptide N-acetylgalactosaminyltransferase 11 (*GALNT11*; rs76030055; *P* = 4.99e−9). In men, HGI was associated with genome-wide significant variants in HECT-type E3 ubiquitin transferase (*HECW2*; rs16849034; *P* = 1.5e−8). These peaks were sex-specific, with minimal associations observed in the opposite sex. **Supplementary Table 4** provides links to interrogatable Manhattan plots for each stratum.

We were unable to find replication cohorts with available data stratified by sex or Hispanic ethnicity. However, we were able to test for replication in ARIC for the self-identified white and black strata. In black participants (ACCORD: *N* = 1,378, ARIC: *N* = 581), the variant *rs10739419* on chromosome 9 in the Whirlin (*WHRN*) gene was formally replicated (**Figures 2D, E**) in ARIC (meta-*P* = 2.20e−9), with each T allele associated with a 0.40 increase in HGI value. In white participants, despite a strong discovery association with HGI in *COL4A2* (*rs9521792*; *P* = 5.17e−8; **Supplementary Figure 1H**) and a consistent direction of effect for all overlapping variants between ARIC and ACCORD, no variants met our criteria for replication in ARIC (**Supplementary Table 5**).

Hispanics are a largely understudied population in human genetics. In the ACCORD cohort, 544 individuals with genotyping data self-identified as Hispanic. We conducted GWAS on these participants. Two discovery loci had genome-wide significant associations with the following sentinel SNPs: *rs2516837* (*P* = 1.48e−9) located on chromosome 1 in the *USF1* gene and *rs141006133* (*P* = 6.92e−9) located upstream of the nucleoredoxin-like 2 (*NXNL2*) gene on chromosome 9 (**Supplementary Figures 1I–K**). All variants for each stratum with *P*<5e−6 are reported in **Supplementary Table 6**.

### 3.4 HGI-associated variants and genes

Variants in any stratum at *P*<5e−6 (**Supplementary Table 6**) were examined for published results in the Human Genome Research Institute GWAS Catalog (30) (downloaded 6.7.2024). The reported traits with HGI-associated variants included height, educational attainment, otosclerosis, platelet count and crit, and alkaline phosphatase (**Supplementary Table 7** *).*

We also investigated the reported traits of HGI-associated genes for genes previously associated with type 2 diabetes. Eight genes associated with HGI were previously associated with type 2 diabetes in the GWAS catalog—*ASAH1*, *CPED1*, *CPNE4*, *JMY*, *PTPRD*, *TMEM132D*, *UBASH3A*, and *UNC13C*. A complete GWAS trait list of HGI-associated genes can be found in **Supplementary Table 8**.

Finally, for the sentinel variants in **Table 2**, we retrieved all variants with LD >0.60 from the GWAS catalog. These results, displayed in **Supplementary Table 9**, highlight numerous blood cell morphometric traits in addition to HbA1c and glomerular filtration rate. Taken together, these results demonstrate that HGI is a heritable trait and that genetic associations with HGI are very different than those with FPG and HbA1c. Consistent with recent publications highlighting the association of HGI with T2D complications and comorbidities, these data suggest the clinical utility of HGI in the management of T2D.

## 4 Discussion

HGI has been associated with the severity of comorbid conditions in individuals with and without type 2 diabetes. In patients with type 2 diabetes, we mapped genetic variants associated with HGI with potential utility in clinical and precision medicine settings. For example, *rs9515223* is a cis-eQTL in the *COL4A2-AS1* RNA gene that has been associated with cerebral small vessel disease and decreased expression of *COL4A2* and *RAB20* in immune cells. This is an interesting discovery as brain atrophy and cerebrovascular disease are associated with type 2 diabetes (31). Additionally, *rs6816425* is a strong cis-eQTL for increased expression of *CXCL11* in the pancreas, which has been implicated in autoimmune disease pathogenesis, including diabetes, and is associated with insulin resistance in obese individuals (32, 33). Of particular interest are variants in and near genes associated with extracellular matrix function and immune function. Genes associated with extracellular matrix function are of interest because influences on collagen synthesis (e.g., variation in lysyl oxidase activity or vitamin C metabolism) represent a potential mechanistic link to the slow loss of vascular integrity and chronic vascular disease, which are associated with diabetes and high HbA1c. Genes associated with immune function are of interest because high HGI has been associated with increased levels of C-reactive protein (10, 13, 34, 35) and fibrinogen (10), which are suggestive of low-grade inflammation and altered systemic redox homeostasis.

The replication of significant findings is necessary to minimize false discovery in GWAS but is potentially problematic, as few substantially sized cohorts measure both HbA1c and FPG. Excitingly, across all ACCORD participants, the lead discovery locus was replicated in ARIC. This variant is an uncharacterized lncRNA. Furthermore, the discovery variant in the *WHRN* gene was replicated in black ARIC participants. Variants in *WHRN* have been associated with Usher syndrome and non-autoimmune adolescent diabetes (36). Variants in *SHLD1* have been associated with multiple traits that include waist-to-hip ratio and apolipoprotein B levels (37, 38). Variants in *FAM222B* have been associated with increased hemoglobin and mean corpuscular hemoglobin (39, 40), along with decreased red blood cell density and erythrocyte count (39, 41).

GWAS trait lookups of all discovery variants from **Supplementary Table 6** are shown in **Supplementary Table 7**. Discovery variants associated with HGI have also been associated with blood protein measurements, body height, platelet crit and count, and alkaline phosphatase measurements (40, 42–45). A lookup of all discovery genes in the associated GWAS catalog (**Supplementary Table 8**) revealed overwhelming associations with blood cell morphometry and quantification traits. We examined all discovery loci in **Table 2** that were listed in the GWAS catalog. This revealed that blood cell morphometry and quantification were overwhelmingly associated with HGI-associated variants (**Supplementary Table 9**). A recent publication highlighted an increase in RBC turnover commensurate with a decrease in HbA1c relative to glucose levels, and an increase in the incidence of diabetes complications (diabetic retinopathy) in individuals with a variant in the *G6PD* gene (5). Including HGI as an additional clinical biomarker would have flagged these individuals for possible glucose management long before indication by aberrant HbA1c measurements, potentially preventing or delaying complications from T2D. Furthermore, we found a strong colocalization signal between HGI variation and expression of PROCA1 in brain, vascular, and pancreatic tissues. Variants in and near *PROCA1* have been associated with cholesterol levels (46) and with reticulocyte count (39), further implicating blood cell counts and morphometric traits in the severity of T2D comorbidities.

In summary, we found sex- and self-reported race differences in association with HGI and achieved local replication in ARIC for the lead locus (rs73397205; CHR7:67736280) in all individuals and a variant in the *WHRN* gene in black individuals. This is striking considering the substantial differences between the ACCORD and ARIC cohorts in terms of participants’ age, BMI, and measures of glucose control, all of which are associated with HGI.

It is critical for research to focus on understudied populations, and we have done so here, although smaller sample sizes resulted in reduced statistical power. Nevertheless, we found striking associations in individuals of Hispanic ancestry. Although we were unable to find a replication cohort for the Hispanic strata in our discovery cohort, we did uncover unique genome-wide significant findings. Genetic mapping of Hispanic individuals in a cohort of this size (*n* = 544) is uncommon, as Hispanic patients are underrepresented in diabetes research, making the results even more important. We discovered genome-wide significant findings in *USF1* and *NXNL2*. *NXNL2* is involved in the sensory perception of smell and sight and is associated with neuroblastoma (47), and *USF1* is a known lipogenic transcription factor (48, 49). Accordingly, follow-up in a cohort of Hispanic ethnicity is warranted.

In the GWAS catalog, HGI-associated variants were largely non-overlapping with FPG and HbA1c associations, two long-established measures of glucose control, indicating that HGI is a distinct, complex genetic trait. Our GWAS results indicate that HGI, like FPG and HbA1c, is highly heritable, with similar heritability to HbA1c (40%–50%). We also found several unique associations in the stratified analyses that indicate heterogeneity by sex and race. For example, we found a genome-wide significant association in women between HGI and *GALNT11*. *GALNT11* plays a role in kidney function deterioration (50), and HGI has been associated with an increased risk of kidney dysfunction (12).

To the best of the authors’ knowledge, this is the first direct genetic study of HGI. We both discovered and replicated a variant locus in a previously characterized lncRNA. Despite substantial differences between ACCORD and ARIC, this locus was replicated in ARIC. Furthermore, we conducted genetic mapping of HGI stratified by self-reported race and sex. Given its emerging clinical importance, the discovery and validation of genetic variants associated with HGI may improve our understanding of its etiology, identify predictive biomarkers, and aid in the identification of increased comorbidities and the discovery of druggable targets.

## Data Availability

Publicly available datasets were analyzed in this study. These data can be found here: https://biolincc.nhlbi.nih.gov/studies/accord/, https://biolincc.nhlbi.nih.gov/studies/aric/.
