# Supplementary material for: A genome-wide association study identifies genetic determinants of hemoglobin glycation index with implications across sex and ethnicity
Source: Front Endocrinol (Lausanne). 2024 Oct 28;15:1473329. doi: 10.3389/fendo.2024.1473329 (PMC11551017; doi:10.3389/fendo.2024.1473329)
Supplement: Supplementary file 1 [file DataSheet1.pdf]

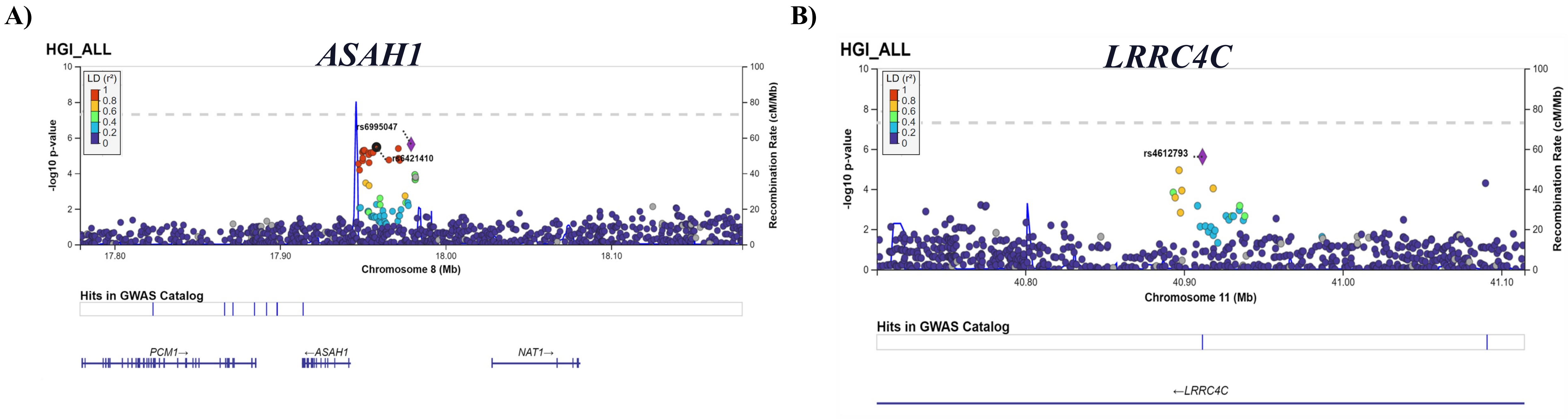

Supplemental Figure 1. Regional Locus Zoom Plots for HGI – All Races and Sex in ACCORD (n = 7913) - A) Regional Locus Zoom plot of the peak and the top two hits in *ASAH1* are labeled and with replication support in ARIC. **B)** The top hit in *LRRC4C* exhibited replication support in ARIC and is labeled.

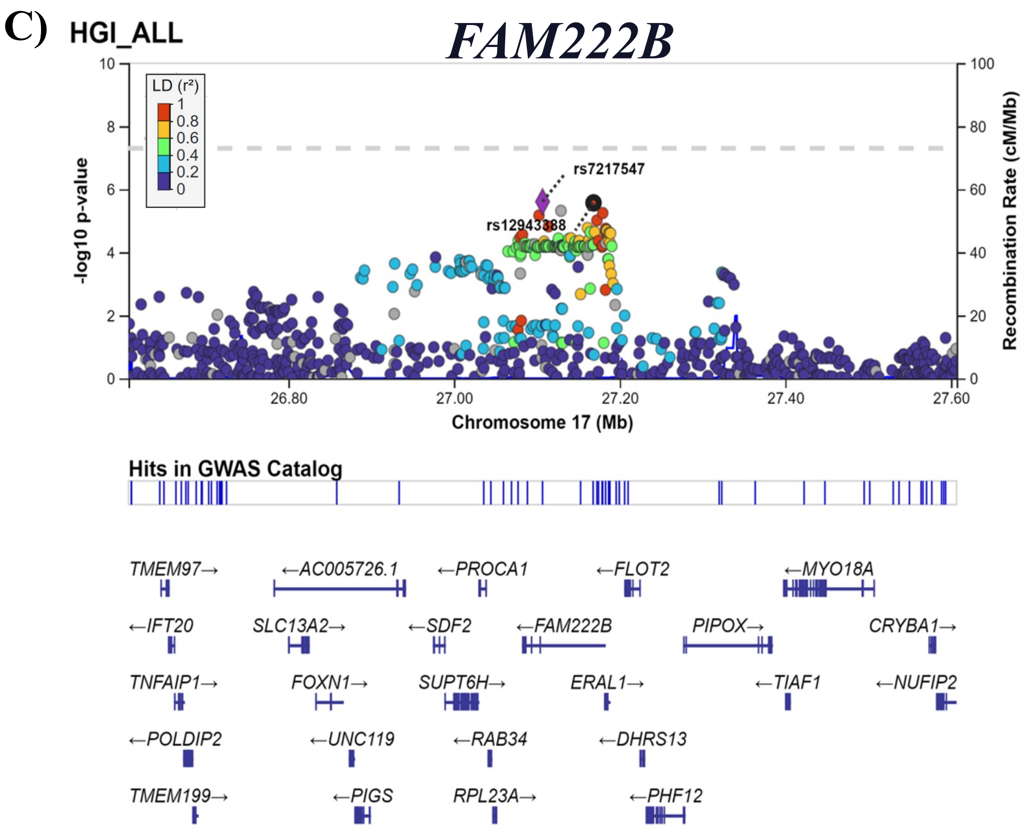

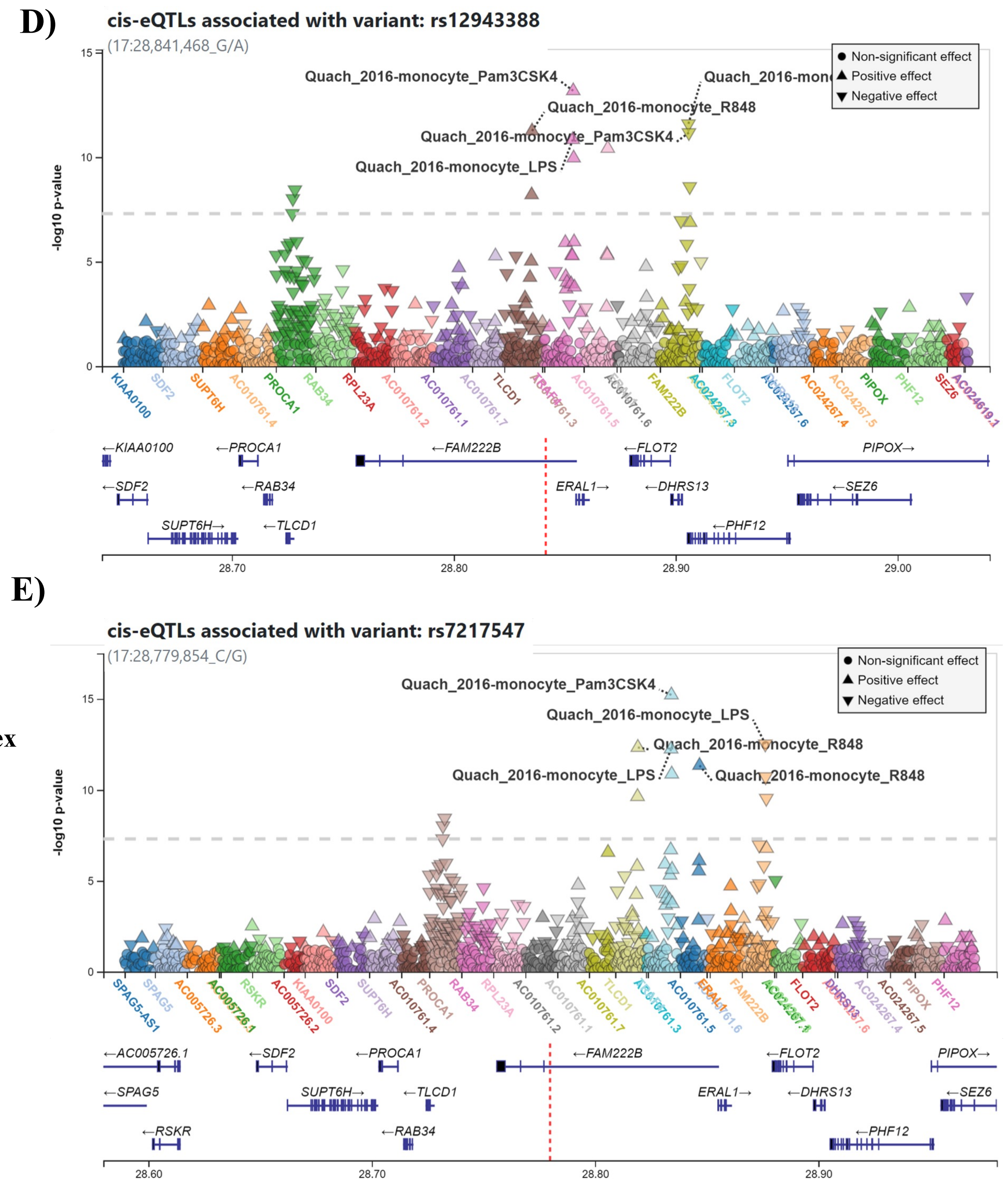

Supplemental Figure 1. Regional Locus Zoom Plots for HGI – All Races and Sex in ACCORD (n = 7913) C) Regional Locus Zoom plot of the peak in *FAM222B*. Strong replication support was found in the top 2 variants (labeled) in ARIC. **D)** Expression quantitative trait loci associated with variant rs12943388 and **E)** rs7217547.

F)

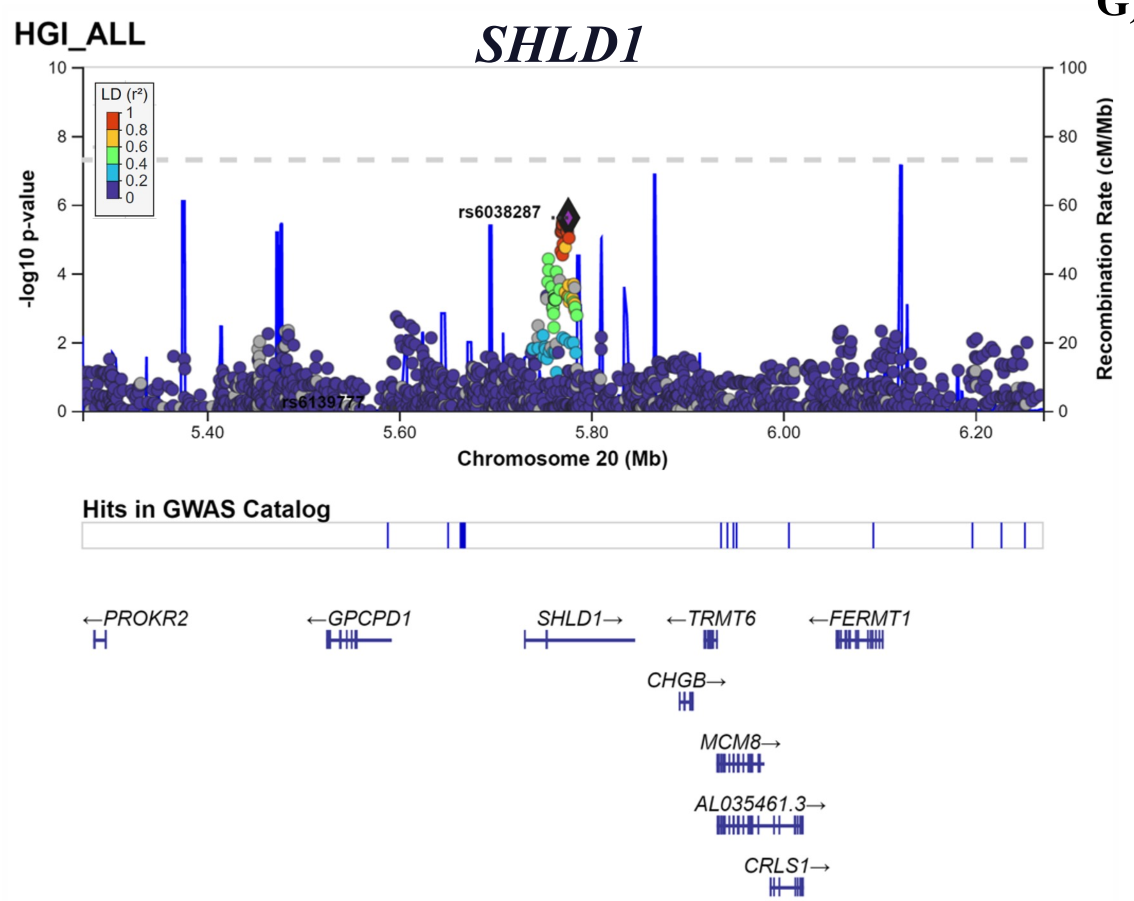

G)

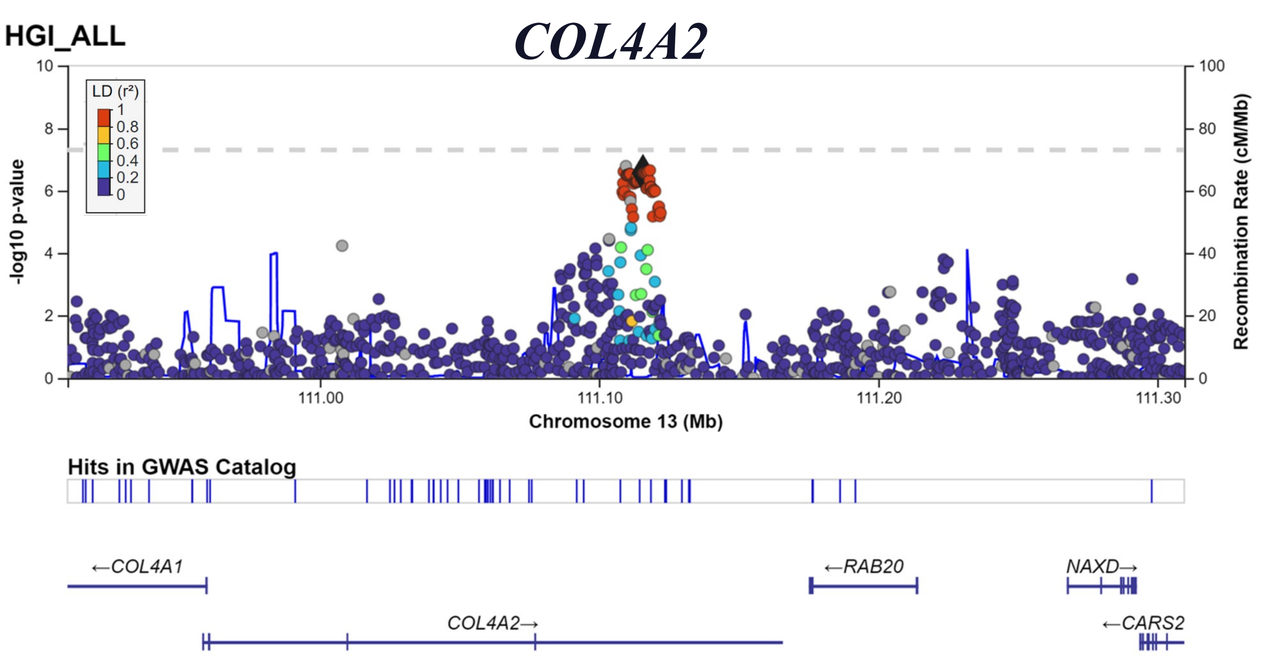

Supplemental Figure 1. Regional Locus Zoom Plots for HGI – All Races and Sex in ACCORD (n = 7913) F) Regional locus zoom plot for *SHLD1* - Strong replication support for 10 variants in ARIC **G)** Regional locus zoom plot for COL4A2 – No replication support in ARIC.

H)

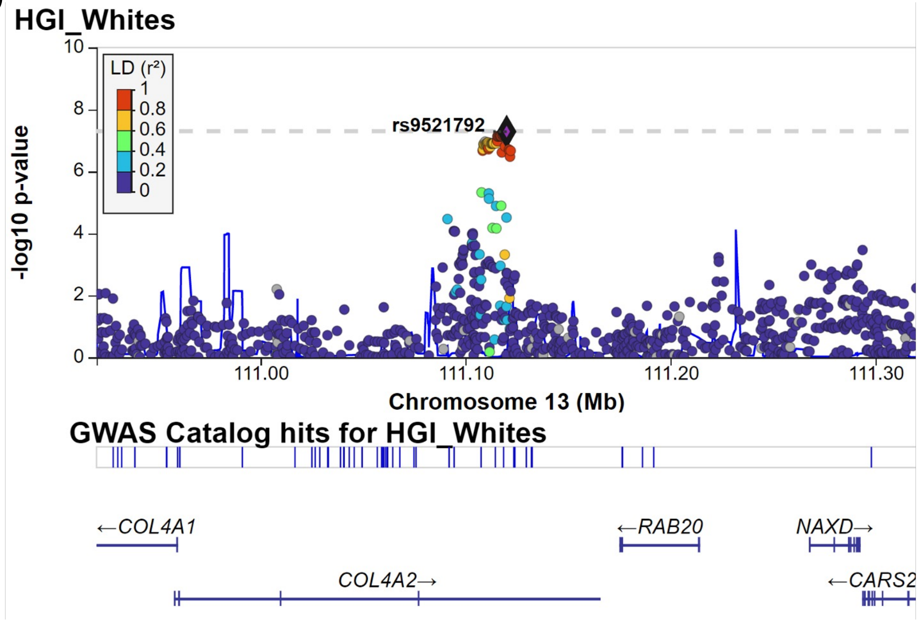

Supplemental Figure 1. Regional Locus Zoom Plots for HGI – whites in ACCORD (n = 5085) H) *COL4A2* peak in whites with lead SNP *rs9521792*. No variants replicated.

I)

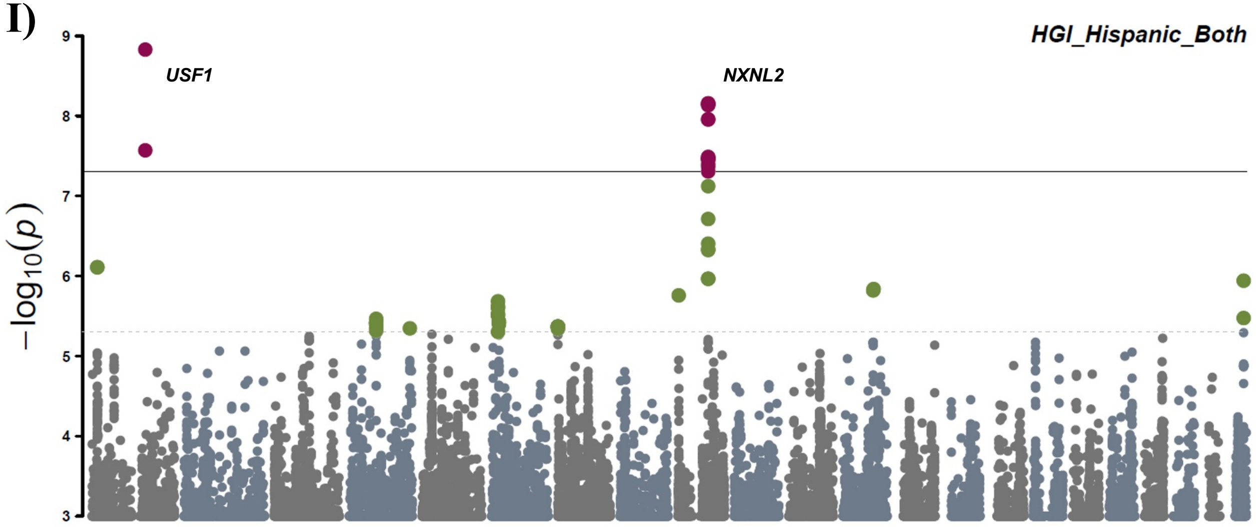

J)

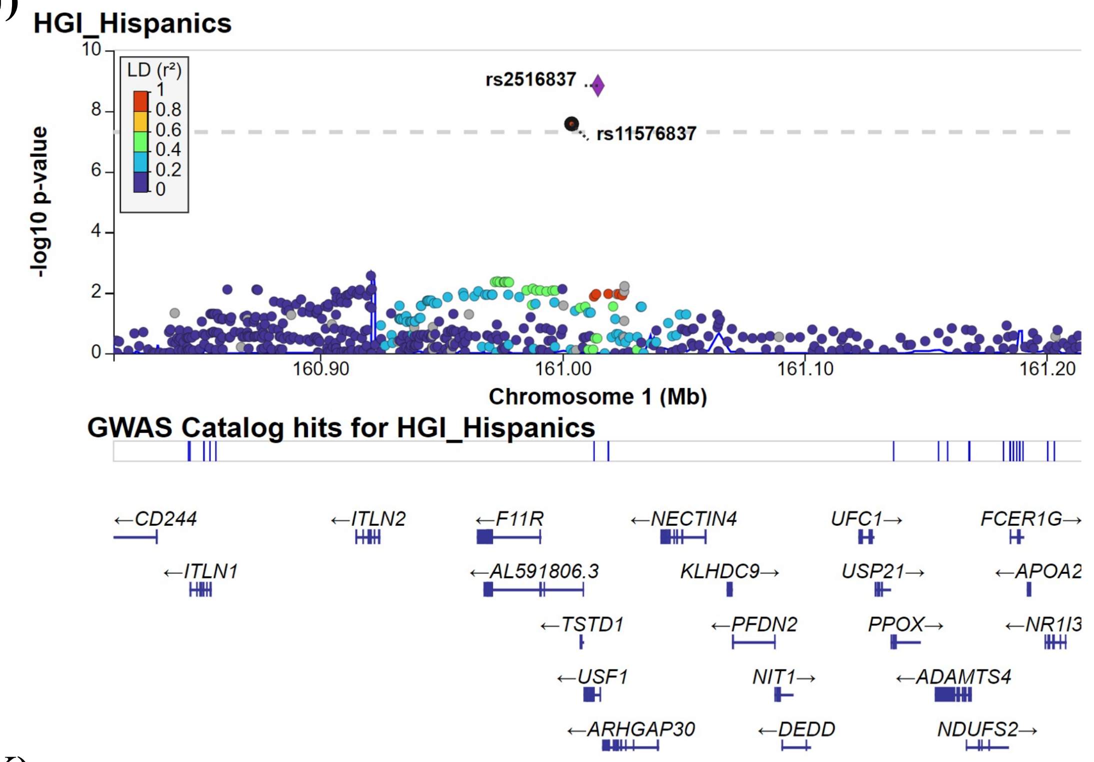

K)

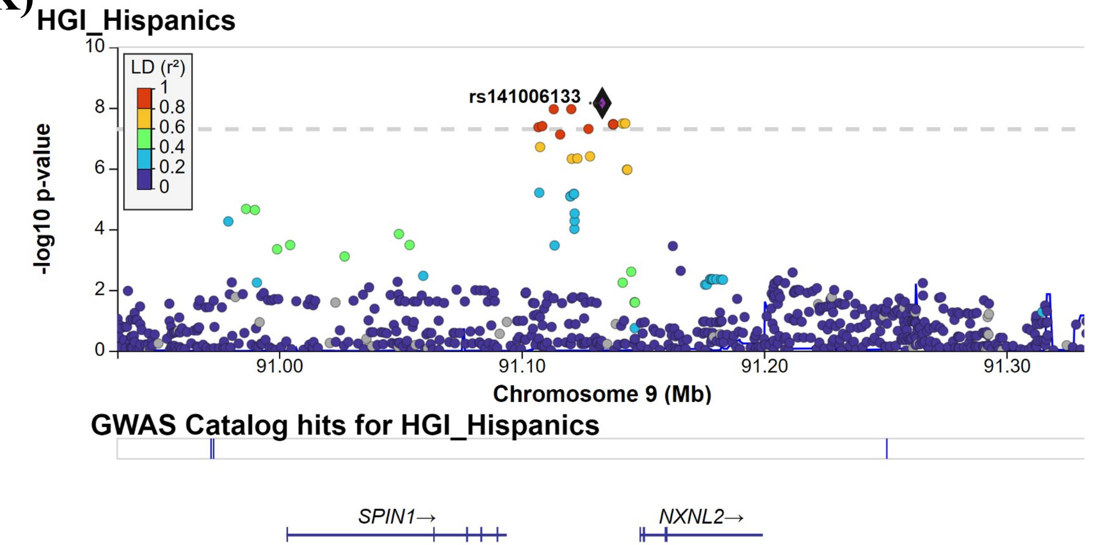

Supplemental Figure 1. Regional Locus Zoom Plots for HGI – Hispanics in ACCORD (n = 544). **I)** Manhattan plot for Hispanics. Genotyped and imputed variants with minor allele frequency > 3% (~ 8 million variants) are ordered by chromosome (X-axis) and –log10(*P-values* < 1e-3) are plotted (Y-axis). *P-values* are adjusted for baseline age, sex, BMI, treatment arm, PC2, and number of years diabetic. Solid line: p = 5e-8; light dotted line p = 5e-6. **J)** Regional Locus Zoom plot of lead signal in promotor region of *USF1*. **K)** Regional Locus Zoom plot of peak upstream of *NXNL2*. LD structure is compared to lead variant and for AMR ancestry.

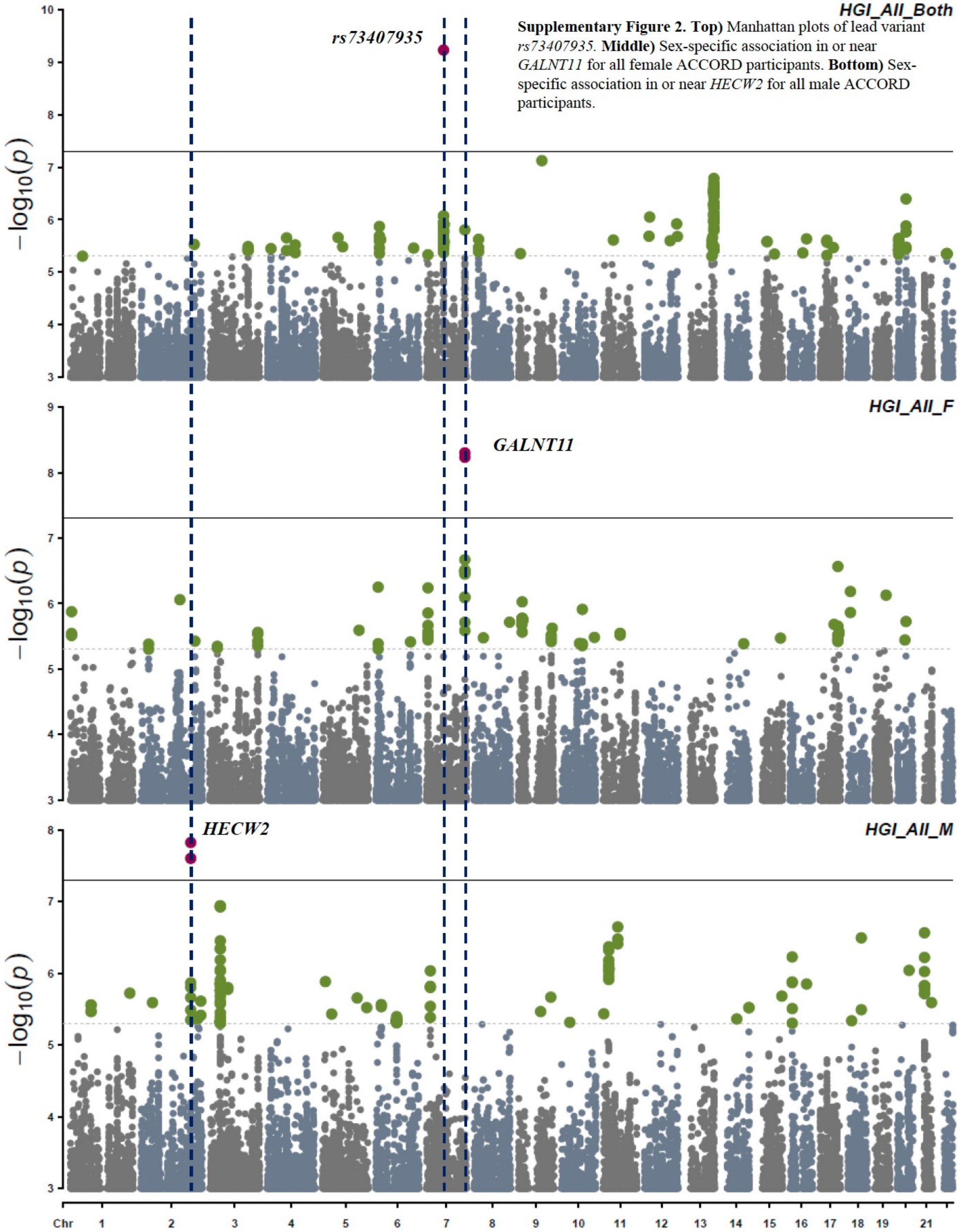

Supplementary Figure 2. Top) Manhattan plots of lead variant *rs73407935*. **Middle)** Sex-specific association in or near *GALNT11* for all female ACCORD participants. **Bottom)** Sex-specific association in or near *HECW2* for all male ACCORD participants.
