# Supplementary material for: A genome-wide association study identifies genetic determinants of hemoglobin glycation index with implications across sex and ethnicity
Source: Front Endocrinol (Lausanne). 2024 Oct 28;15:1473329. doi: 10.3389/fendo.2024.1473329 (PMC11551017; doi:10.3389/fendo.2024.1473329)
Supplement: Supplementary file 2 [file DataSheet2.docx]

Supplementary Material

# Supplementary Methods

The landmark ACCORD trial included 10,251 participants with type 2 diabetes who had experienced or had other evidence of high risk for a cardiovascular event with a primary endpoint of time to first occurrence of a major adverse cardiovascular event (MACE), namely non-fatal myocardial infarction, non-fatal stroke, or cardiovascular death. Median follow-up time was 5·0 years (mean 5·0; range 0·01–8·4). ACCORD findings have important implications and have helped shape current glycemic management guidelines (1-3). The rationale, study design, inclusion criteria, and other details of the ACCORD trial are described elsewhere.

The ACCORD trial compared the effects of intensive glycemia treatment with a target of glycated hemoglobin A1c (HbA1c) < 6% and standard treatment with a target of HbA1c=7·0-7·9%. Despite a significant increase in mortality in the intensive treatment arm, which led to its early termination (4), heterogeneity in treatment response was observed (5-8). Individuals who received intensive treatment but did not reach the HbA1c target of 7·0-7·9% had the greatest risk of mortality and MACE (9). ACCORD participants in the intensive treatment arm in the high HGI tertile had a 41% increase in total mortality but no change in risk of the primary endpoint (i.e., the first occurrence of myocardial infarction, stroke, or cardiovascular death). In contrast, participants in the lower and middle HGI tertiles had no increase in mortality but an approximately 24% decreased risk of the primary endpoint (7).

The multicenter, randomized clinical trial used a double 2 × 2 factorial design to incorporate intervention trials for glycemia, hyperlipidemia, and hypertension. With a sample of 5,518 participants, the lipid intervention trial tested the hypothesis that a hypolipidemic agent would improve outcomes in participants with good glycemic control. With a sample of the remaining 4,733 participants, the blood pressure intervention trial tested the hypothesis that a therapeutic strategy with a target systolic blood pressure of < 120 mmHg would reduce the rate of cardiovascular events compared to a strategy with a target systolic blood pressure of < 140 mmHg.

The glycemia intervention trial included all 10,251 ACCORD participants, who were randomly assigned at baseline to a standard or intensive treatment arm. Participants were permitted to use any antihyperglycemic agent or combination of agents approved by regulatory authorities to achieve target HbA1c levels=7·0-7·9% (53-63 mmol/mol) in the standard treatment arm or < 6·0% (42 mmol/mol) in the intensive treatment arm. Participants in the intensive treatment group were converted to the standard treatment regimen after 3·5 years due to observed excess mortality (4). For the present study, we obtained phenotype data on all ACCORD participants but used data only from participants with FPG and HbA1c recorded at baseline who consented to genotyping in our analyses (n = 7,913).

**Genotyping**

The University of Washington extracted genomic DNA from white cell preparations using the FlexiGene DNA Kit (Qiagen, Valencia, CA) following the manufacturer’s instructions. The picogreen dye labeling method (Quant-iT, Invitrogen, Eugene, OR), diluted in Tris-EDTA buffer and plated into DNAse/RNAse free multiwall plates, was used for double-stranded DNA quantification. Two independent laboratories performed genome-wide genotyping on different platforms. The University of Virginia genotyped 6,085 unique samples from ACCORD participants who consented to inclusion in genetic studies conducted by any investigator on Illumina HumanOmniExpressExome-8 1·0 chips (Set 1). The University of North Carolina genotyped 8,174 unique samples, comprising the 6,085 samples above plus 2,089 samples from ACCORD participants who consented only to genetic studies conducted by ACCORD investigators, on Affymetrix Axiom Biobank1 chips (Set 2). As described in detail in Marvel and Rotroff et al. (10), genotypes in Set 1 and Set 2 were subjected to rigorous quality control based on genotyping quality metrics, including sample/marker missingness, predicted gender, cryptic relatedness, duplicate concordance, and Hardy-Weinberg equilibrium.

Sets 1 and 2 were subsequently merged to create a single dataset comprising 5,971 genotyped samples with 1,240,656 individual SNPs and an additional 2,083 genotyped samples with 583,613 SNPs. The genotyping data from all participants (N = 8,174) were genotyped on the UNC Affymetrix array were imputed to 26.3 million SNPs (after removing imputed variants with INFO score < 0.5) using a two-step approach. The genotype calls were first pre-phased using SHAPEIT2 (v2·r778) and then imputed using IMPUTE2 (v2·3.0) (10, 11). The integrated haplotypes reference panel from Phase 1 of the 1000 Genomes (release date Dec 2013) from the IMPUTE2 website was used for both steps.

1. Garber AJ, Abrahamson MJ, Barzilay JI, Blonde L, Bloomgarden ZT, Bush MA, et al. Consensus statement by the American Association of Clinical Endocrinologists and American College of Endocrinology on the comprehensive type 2 diabetes management algorithm. Endocr Pract. 2019;25(1):69-100.

2. American Diabetes Association. Glycemic Targets: Standards of Medical Care in Diabetes-2019. Diabetes Care. 2019;42(Suppl 1):S61-s70.

3. Genuth S, Ismail-Beigi F. Clinical implications of the ACCORD trial. J Clin Endocrinol Metab. 2012;97(1):41-8.

4. Gerstein HC, Miller ME, Byington RP, Goff DC, Jr., Bigger JT, Buse JB, et al. Effects of intensive glucose lowering in type 2 diabetes. N Engl J Med. 2008;358(24):2545-59.

5. Shah HS, Gao H, Morieri ML, Skupien J, Marvel S, Paré G, et al. Genetic predictors of cardiovascular mortality during intensive glycemic control in type 2 diabetes: Findings from the ACCORD Clinical Trial. Diabetes Care. 2016;39(11):1915-24.

6. Basu S, Raghavan S, Wexler DJ, Berkowitz SA. Characteristics Associated With Decreased or Increased Mortality Risk From Glycemic Therapy Among Patients With Type 2 Diabetes and High Cardiovascular Risk: Machine Learning Analysis of the ACCORD Trial. Diabetes Care. 2018;41(3):604-12.

7. Hempe JM, Liu S, Myers L, McCarter RJ, Buse JB, Fonseca V. The hemoglobin glycation index identifies subpopulations with harms or benefits from intensive treatment in the ACCORD trial. Diabetes Care. 2015;38(6):1067-74.

8. Riddle MC, Ambrosius WT, Brillon DJ, Buse JB, Byington RP, Cohen RM, et al. Epidemiologic relationships between A1C and all-cause mortality during a median 3.4-year follow-up of glycemic treatment in the ACCORD trial. Diabetes Care. 2010;33(5):983-90.

9. Riddle MC, Karl DM. Individualizing targets and tactics for high-risk patients with type 2 diabetes: practical lessons from ACCORD and other cardiovascular trials. Diabetes Care. 2012;35(10):2100-7.

10. Marvel SW, Rotroff DM, Wagner MJ, Buse JB, Havener TM, McLeod HL, et al. Common and rare genetic markers of lipid variation in subjects with type 2 diabetes from the ACCORD clinical trial. PeerJ. 2017;5:e3187.

11. Irvin MR, Rotroff DM, Aslibekyan S, Zhi D, Hidalgo B, Motsinger-Reif A, et al. A genome-wide study of lipid response to fenofibrate in Caucasians: a combined analysis of the GOLDN and ACCORD studies. Pharmacogenet Genomics. 2016;26(7):324-33.
